# Supplementary prenatal copper increases plasma triiodothyronine and brown adipose tissue uncoupling protein-1 gene expression but depresses thermogenesis in newborn lambs

**DOI:** 10.5713/ajas.18.0179

**Published:** 2019-05-28

**Authors:** Stephen B. Smith, Craig R. Sweatt, Gordon E. Carstens

**Affiliations:** 1 Department of Animal Science, Texas A&M University, College Station, TX 77843, USA

**Keywords:** Brown Adipose Tissue, Copper, Lambs, Thermogenesis, Uncoupling Protein-1

## Abstract

**Objective:**

We tested the hypothesis that increasing dietary copper (Cu) to gravid ewes would enhance brown adipose tissue (BAT) thermogenesis in their offspring.

**Methods:**

Twin-bearing ewes were assigned on d 70 of gestation to diets containing 3, 10, or 20 ppm dietary Cu (n = 8 per group). Twin lambs were assigned at birth to a cold (6°C) or warm (28°C) environmental chamber for 48 h. Blood was collected from ewes and from lambs and perirenal BAT was collected after 48 h in the environmental chambers.

**Results:**

Prenatal Cu exposure increased ewe plasma triiodothyronine (T_3_) and thyroxine concentration (T_4_) (p<0.01) but prenatal Cu exposure had no effect on lamb plasma concentrations of T_3_, T_4_, glucose, or nonesterified fatty acid concentration (p≥0.08). The high level of prenatal Cu exposure depressed 48-h rectal temperature (p = 0.03). Cold exposure decreased BAT norepinephrine (NE) and increased BAT dopamine (p≤0.01), but prenatal Cu exposure had no effect on BAT cytochrome C oxidase activity or BAT NE or dopamine (p≥0.07). However, BAT of lambs from high-Cu ewes maintained higher uncoupling protein-1 (*UCP1*) gene expression than BAT of lambs from low- and medium-Cu ewes following warm or cold exposure in environmental chambers (p = 0.02). Cold exposure caused near depletion of BAT lipid by 48 h (p<0.001), increased BAT cytochrome c oxidase activity (p< 0.01), and depressed plasma fatty acid concentrations (p<0.001).

**Conclusion:**

Although prenatal Cu exposure increased BAT *UCP1* expression during warm and cold exposure, prenatal cold Cu exposure depressed 48-h rectal temperature. Cold exposure decreased BAT lipid content by over 80% and decreased lamb plasma fatty acid concentration by over 40%, indicating that fuel reserves for thermogenesis were nearly depleted by 48 h of cold exposure.

## INTRODUCTION

Approximately one-half of maximal thermogenic response to cold stress in newborn lambs is derived from nonshivering thermogenesis of brown adipose tissue (BAT) [1]. The ability of BAT to generate heat is due to the presence of uncoupling protein-1 (UCP1), found only in BAT mitochondria. When stimulated by cold exposure, UCP1 acts to uncouple oxidative phosphorylation from fatty acid oxidation, thereby causing BAT mitochondria to generate heat rather than ATP. Brown adipose tissue is highly innervated by the sympathetic nervous system [2] which, during cold exposure, releases norepinephrine (NE) to activate BAT thermogenesis by stimulating fatty acid oxidation and *UCP1* gene expression. Additionally, NE activates type II thyroxine 5’deiodinase in BAT to convert thyroxine (T_4_) to the more active thyroid hormone triiodothyronine (T_3_) [3]. Local synthesis of T_3_ is also an important regulator of *UCP1* gene expression [4]. Thus, both NE and T_3_ play a critical role in regulating BAT thermogenesis in newborn lambs.

Eales et al [5] reported that newborn lambs that were hypo thermic due to cold exposure had low plasma Cu concentrations (0.18 μg/mL) compared to plasma Cu concentrations in lambs that did not display hypothermia (0.63 μg/mL), which suggests that maternal Cu deficiency may impair the cold tolerance of newborn lambs. There are several Cu-dependent enzyme systems that play critical roles in regulating BAT thermogenesis, including cytochrome c oxidase (COX; electron transport system) and dopamine-β-hydroxylase (conversion of dopamine to NE in the sympathetic nervous system). Additionally, Lukaski et al [6] reported that Cu deficiency in rats reduced plasma T_3_ and T_4_ concentrations and reduced BAT deiodinase activity, resulting in hypothermia. Therefore, we hypothesized that increasing dietary Cu to gravid ewes would enhance BAT thermogenesis in their offspring.

## MATERIALS AND METHODS

### Ethics statement

The experimental procedures were approved by the Texas A&M University Animal Care and Use Committee of the Office of Research Compliance.

### Animals and diets

Twenty-four Rambouillet ewes were determined to be twin-bearing by ultrasound during the first trimester of gestation. Ewes were housed in an open-sided barn, and provided free access to fresh water and coastal bermudagrass hay containing 0.76 mg Cu/kg DM. Ewes were adapted to a high-concentrate diet for 3 wk (Table 1, without supplemental S, Mo, or Cu) that was formulated to meet NRC requirements [7], with the exception of Cu. Thereafter, ewes were assigned on approximately d 50 of gestation to 1 of 3 diets containing 3, 10, or 20 ppm dietary Cu (n = 8 lambs per Cu concentration). Additionally, each diet contained 2 ppm (DM basis) Mo and 0.28% S to limit the absorption of dietary Cu. Accounting for the dietary concentrations of Mo and S, the net available Cu concentrations [8] were 0.12, 0.36 and 0.72 ppm (DM basis) for the low-, medium- and high-Cu diets, respectively. The medium-Cu treatment was formulated to meet the estimated net available Cu requirements for 75 kg twin-bearing ewes, which is 0.63 mg Cu/d [8]. Each prenatal Cu treatment consisted of 4 ewes/pen and 2 pens/treatment. Ewes were fed individually in an open-sided barn. Food intake and body weight were measured at 7-d intervals. Food intake was calculated as total food provided minus food remaining.

Ewes were monitored at 4-h intervals prior to expected lambing. Immediately following parturition, lambs were separated from ewes, dried of amniotic fluid, weighed, and moved to a warm chamber (25°C). Initial blood samples were obtained by venipuncutre and lambs were fed 38 mL/kg BW pooled bovine colostrum. At 2 h of age, lambs were assigned randomly within twin pair to cold (6°C) or warm (28°C) environmental chambers for 48 h. At 8, 14, and 20 h of age, lambs were fed 30 mL/kg BW colostrum. Rectal temperatures were measured at 48 h. Blood was collected obtained by jugular venipuncture in heparinized vacutainer tubes from ewes at 7 d prior to birth between 0700 and 0800 before feeding and from lambs prior to slaughter at 48 h of age, and plasma was analyzed for Cu, ceruloplasmin, T_3_, T_4_, and cortisol. Glucose and nonesterified fatty acids (NEFA) also were measured in lamb plasma. Blood samples were obtained by jugular venipuncture in heparinized vacutainer tubes between 0700 and 0800, before feeding. Equal volumes of 0.9% saline were infused to replace blood volume.

### Collection of brown adipose tissue

Following the 48-h environmental-chamber period, lambs were administered an overdose of sodium pentobarbital and exsanguinated, and perirenal BAT was removed completely and weighed. A portion of the BAT was snap-frozen in liquid nitrogen and stored at −80°C.

### Histological analysis

Small, unfrozen samples of BAT (~100 mg) were prepared for histological analysis. Tissue samples were sliced into 1-mm size pieces and placed into a primary fixative of 3% glutaraldehyde:0.08 *M* sodium cacodylate buffer (pH 7.4). One perirenal BAT sample from a low-Cu, warm-chamber lamb, and one sample from a high-Cu, cold-chamber lamb were post-fixed in 20 g/L osmium tetroxide, stained with enbloc stain (2% uranylacetate in methanol), and embedded in Epon/Araldite [9]. The embedded samples were sectioned to approximately 70-nm thickness and photographed at 60 kV with a transmission electron microscope (Zeiss 10 C, Germany).

### Preparation and analysis of RNA

Total RNA was isolated from previously snap-frozen perirenal BAT as described previously [10]. Purity and yield were determined by the ratio of absorbances at 260 and 280 nm, which exceed 1.8 for all samples. A *UCP1* cDNA was generated by polymerase chain reaction. The template DNA was the bovine calf *UCP1* 1.4-kb cDNA (generously provided by L. Casteilla, Centre de Rechere, CNRS, France) linearized with EcoR1. The primers were 5′-CTC AGC GGG CCT AAC GAC-3′ and 5′-GTT TGT TTT TCA CCA GGG-3′. *UCP1* mRNA was determined by slot blot analysis as described previously [10].

### Isolation of mitochondria

Mitochondria were isolated by differential centrifugation as described by Cannon and Lindberg [11]. Fresh, unfrozen BAT samples were dissected and washed immediately in sucrose buffer (250 mmol/L sucrose, 5 mmol/L TES-HCl, pH 7.2). BAT samples were homogenized in 10 volumes (wt/vol) of sucrose buffer and centrifuged at 800×*g* for 10 min. The pellet was resuspended to original volume in fresh sucrose buffer. The suspension was centrifuged at 800×*g* for 10 min to sediment nuclei and cellular debris, and the supernate was centrifuged at 10,000×*g* for 10 min. The mitochondria pellet was resuspended to 0.5 mL in fresh sucrose buffer. Aliquots of homogenate and mitochondrial preparations were frozen at −80°C for subsequent determination of COX activity [12], DNA [13], protein [14], and lipid [10]. Total mitochondrial protein was determined based on mitochondrial recovery from preparations.

### Plasma Cu, hormones, and nonesterified fatty acids

Plasma Cu was determined by flame atomic absorption spectrophotometry (Model S 11, Thermo Jarrell Ash, Franklin, MA, USA). Blood samples were centrifuged at 2,000×g and plasma harvested and stored at −20°C. Plasma ceruloplasmin activity was measured as described previously [15]. Plasma NEFA were measured with a commercial kit (Wako Chemicals, Brentwood, NY, USA). Concentrations of T_3_, T_4_, and cortisol were measured using commercial radioimmunoassay kits (Pantex, Santa Monica, CA, USA). Dopamine and NE were measured by high-performance liquid chromatography [16].

### Source of chemicals

Unless otherwise stated, biochemicals were purchased from Sigma Chemical (St. Louis, MO, USA) and Gibco BRL (Gaithersburg, MD, USA). Radiolabeled materials were obtained from Amersham (Arlington Heights, IL, USA).

### Analysis of data

The data were analyzed using the Proc Mixed model for analysis of variance to obtain main effects means, subclass means, and standard errors [17]. For ewe performance data, fixed effect was Cu treatment and random effects were ewe and pen; pen was the experimental unit. For lamb data, fixed effects were prenatal Cu treatment and chamber temperature and random effect was lamb. Lambs were housed individually in chambers and individual lamb was the experimental until. The three-way interaction of prenatal dietary Cu (low, medium, and high), chamber temperature (6°C and 25°C) and time in chamber (0 and 48 h) was not significant for rectal temperature, body weight, or any plasma dependent variables. Therefore, the data for lamb body weight, rectal temperature, and lamb plasma dependent variables (as well as BAT dependent variables) were analyzed as a 2×2 factorial with prenatal dietary Cu and chamber temperature as the main effects; the model also tested the Cu×chamber temperature two-way interactions.

## RESULTS

### Ewes

There was no difference in initial or final body weight, average daily again, or dry matter intake in ewes in response to increasing concentrations of dietary Cu (p≥0.19; Table 2). Dietary Cu treatment had no effect on plasma ceruloplasmin activity and Cu concentration in ewes (p≥0.11) (Table 3). Plasma T_3_ and T_4_ concentrations were higher (p<0.01) in ewes fed the high-Cu diet than in ewes fed the low-Cu diet.

### Effects of prenatal Cu and cold exposure on lambs

Chamber temperature had no effect on plasma Cu concentration or ceruloplasmin activity (p>0.25), so data for these variables were pooled between warm and cold chamber temperatures (Table 3). At birth, there were no differences among prenatal Cu treatments for Cu concentration or ceruloplasmin activity (p>0.25). Plasma ceruloplasmin activity increased with age for all prenatal Cu treatment groups (p<0.001), and ceruloplasmin activity was greater in lambs from high-Cu ewes than in lambs from low-Cu ewes (p = 0.05).

Prenatal Cu exposure, chamber temperature, and the Cu× temperature interaction were not significant for lamb body weight (p>0.25) (Table 4). Rectal temperature at birth (38.6°C ±0.31°C) was not different among prenatal Cu treatments (p >0.25; data not shown). Rectal temperature was lower (p< 0.001) in cold-exposed than in warm-exposed lambs and the high level of prenatal Cu exposure depressed rectal temperature (p = 0.03) (Table 4).

Lamb plasma T _3_ concentration tended (p = 0.08) to decrease as prenatal Cu level increased (Table 4) and plasma T_3_ concentration was greater (p<0.001) in cold-exposed lambs than in warm-exposed lambs. Lamb plasma T_4_ concentration was unaffected by prentatal Cu or environmental temperature (p>0.25). Lamb plasma cortisol concentration was higher (p<0.001) in cold-exposed than warm-exposed lambs. The cold-induced increase in plasma cortisol concentrations was greater in lambs born to high-Cu ewes than in lambs born to low-Cu ewes (prenatal Cu×temperature interaction p = 0.05). Cold-exposed lambs had lesser plasma NEFA concentration than warm-exposed lambs (p<0.001). Plasma glucose concentration was unaffected (p>0.25) by prenatal Cu or postnatal temperature treatments.

Postnatal cold exposure decreased (p <0.001) BAT mass and lipid concentration and increased (p<0.01) BAT protein and moisture concentration, mitochondrial protein concentration, and cytochrome c oxidase activity (Table 5), regardless of prenatal Cu treatment. Postnatal cold exposure decreased (p<0.01) BAT NE concentration and increased (p<0.001) BAT dopamine concentration. The increase in BAT dopamine concentration was significant only for lambs born to high-Cu ewes (prenatal Cu×temperature interaction p = 0.04).

**Figure 1:**
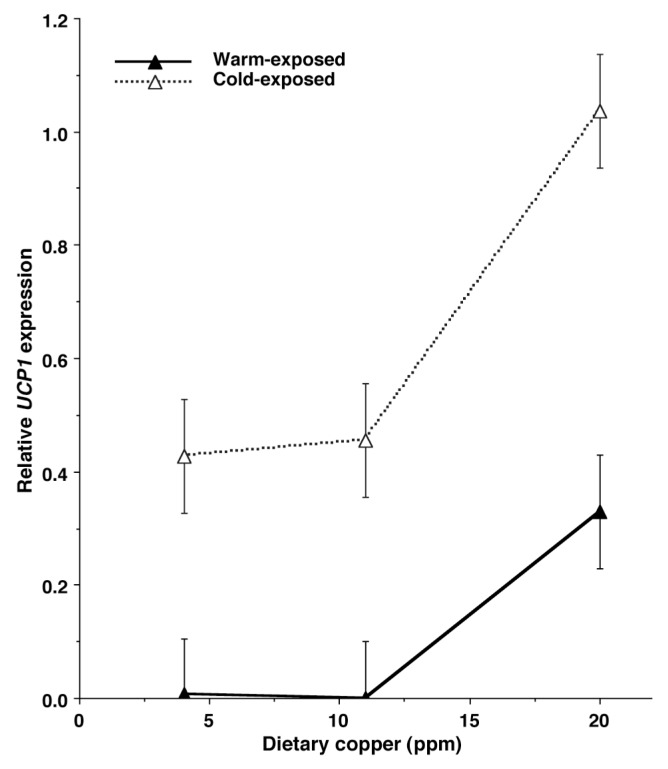
Relative *UCP1* expression for mRNA from brown adipose tissue of lambs from ewes fed low (3.2 ppm), medium (10 ppm), or high (20 ppm) Cu during the last two trimesters of gestation. *UCP1*, uncoupling protein-1. Lambs were held at 6°C (cold) or 28°C (warm) temperatures for 48 h postnatally. Prenatal Cu effect p = 0.02; postnatal temperature effect (warm vs cold) p<0.001; prenatal Cu X postnatal temperature interaction p>0.25. Pooled standard error bars for each treatment are attached to the symbols.

Relative *UCP1* gene expression was higher (p<0.001) in BAT from cold-exposed lambs than in BAT from warm-exposed lambs; *UCP1* gene expression was virtually undetectable in BAT from lambs held at 28°C, except for BAT from high-Cu lambs (Figure 1). *UCP1* gene expression was higher in BAT of lambs from high-Cu ewes than in BAT of lambs from low- and medium-Cu ewes (p = 0.02). The increase in *UCP1* gene expression due to cold exposure was not affected by prenatal Cu treatment (prenatal Cu×chamber temperature interaction p>0.25).

### Lamb brown adipose tissue morphology

**Figure 2:**
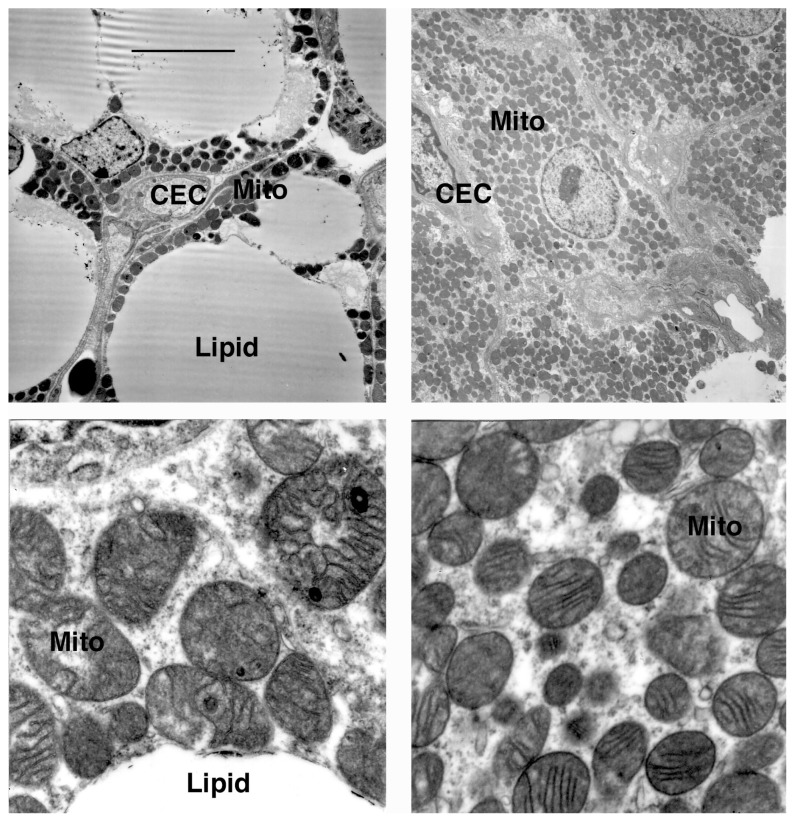
Electron microscopy of perirenal brown adipose tissue from newborn lambs exposed to 28°C (left) or 6°C (right) for 48 h postnatally. CEC, capillary endothelial cell; M, mitochondrion; lipid, lipid vacuole. Top, ×2,500; bottom, ×25,000. Scale bar in the upper left panel indicates 50 μm at 2,500×magnification

Transmission electron micrographs were generated only for the two temperature/Cu extremes, i.e., warm-exposed lambs from low-Cu ewes and cold-exposed lambs from high-Cu ewes. Brown adipocytes from warm-exposed lambs contained large, central lipid droplets, whereas brown adipocytes from cold-exposed lambs contained virtually no visible lipid droplets (Figure 2). The cristae of mitochondria from cold-exposed lambs were more evident than cristae in mitochondria of warm-exposed lambs.

## DISCUSSION

An early report [5] suggested that increasing maternal dietary Cu may attenuate hypothermia in lambs during cold exposure. Because several Cu-dependent enzyme systems are involved in regulating BAT thermogenesis, including COX and dopamine-β-hydroxylase, we hypothesized that increasing Cu intake in gravid ewes would improve cold tolerance in newborn lamb. However, opposite to our hypothesis, increased Cu intake by ewes resulted in more rapid heat loss in cold-exposed lambs, which may have been caused by increased *UCP1* expression in lambs from high-Cu ewes.

### Copper status

The net availabilities of Cu were estimated to be 0.12, 0.36, and 0.72 mg Cu/kg DM for the low-, medium- and high-Cu prenatal treatments, respectively [8]. Based on ewe dry matter intake, net Cu intakes for the three treatments were 0.21, 0.62, and 1.22 mg/d, respectively. Estimated net Cu requirements for 75-kg twin-bearing ewes during late gestation are 0.63 mg Cu/d [8]. Thus, dietary Cu concentration was adequate for the medium-Cu ewes, and deficient and excessive for low- and high-Cu ewes, respectively. However, the prenatal Cu treatments had no effect on plasma Cu concentration of the ewes and lambs, which would suggest that the ewes likely had adequate hepatic Cu stores prior to entry into the study. Previous research [18] demonstrated that plasma Cu concentration and ceruloplasmin activity are low at birth and increase early postnatally, and the current study also demonstrated a postnatal increase in ceruloplasmin activity. Thus, by all measures, neither the ewes fed the low-Cu diets nor their lambs were Cu-deficient.

### Thyroid hormones and brown adipose tissue thermogenesis

Rapid accumulation of lipid in fetal lamb perirenal BAT occurs from d 70 to 120, whereas increased mitochondrial biogenesis and sympathetic innervation of BAT occurs thereafter until parturition at d 150 [19]; this was confirmed in bovine BAT [20]. Relative *UCP1* expression in ovine perirenal BAT reaches a peak at birth and then declines rapidly postnatally in lambs [21] and calves [20].

Giralt et al [22] examined ontogenic changes in type II thyroxine 5′-deiodinase activity in bovine BAT, which first appeared in fetal perirenal BAT at d 60 of gestation and increased rapidly until reaching a peak at d 210 of gestation. Giralt et al [22] hypothesized that endogenous production of T_3_ may be involved in prenatal induction of *UCP1* expression in BAT; this subsequently was confirmed [23].

Treating rats with T _3_ for 5 d and then holding them at 22°C increased *UCP1* expression in interscapular BAT [24]. Cold exposure increased interscapular BAT *UCP1* expression, but under these conditions, T_3_ did not increase *UCP1* expression further. In the current study, lambs from ewes fed the lowest supplemental Cu had the highest rectal temperatures and concomitantly the highest plasma T_3_. However, *UCP1* expression was highest in lambs from ewes fed the high-Cu diets and in fact, *UCP1* expression in BAT from the warm-exposed lambs was detectable only in BAT of calves from high-Cu ewes. Furthermore, the electron micrographs suggest loss of cristae integrity in BAT of lambs from warm-exposed ewes. Thus, circulating T_3_ concentrations in the lambs may have been responsible for the elevated rectal temperatures in the low-Cu lambs, but did not appear to regulate *UCP1* gene expression.

### Norepinephrine and brown adipose tissue thermogenesis

One of the key changes that occurs in BAT late in gestation is the development of sympathetic innervation [25], which increases the activity of thyroxine 5′-deiodinase [26,27]. In addition to its role in acute activation of BAT thermogenesis, NE also is involved in long-term modulation of BAT growth and development during cold stress by enhancing differentiation of BAT precursor cells, mitochondrial proliferation, and *UCP1* expression via β_3_- and α_1_-adrenergic receptor pathways [28]. Norepinephrine increases the activity of type II thyroxine 5′-deiodinase and thereby promotes local production T_3_, which in turn increases *UCP1* expression in BAT [26].

Consistent with the higher plasma T _3_ concentration in low-Cu lambs, NE concentration also were higher in BAT from the low-Cu lambs. Unexpectedly, NE concentration was higher, and dopamine concentration was lower, in BAT from warm-exposed lambs than in BAT from cold-exposed lambs. This may have been in response to increased NE secretion caused by elevated sympathetic stimulation.

### Cold exposure and brown adipose tissue thermogenesis

We have investigated the postnatal changes in perirenal BAT in neonatal calves, and reported that COX activity of perirenal BAT was highest at birth and decreased substantially after 7 d of warm exposure [29]. Trayhurn et al [30] reported a similar postnatal decline in COX activity in warm-exposed, neonatal goats. The results of the current study confirm earlier observations, in that COX activity was approximately threefold higher in BAT of cold-exposed lambs than in warm-exposed lambs. However, COX activity was unaffected by prenatal Cu supplementation.

Especially noteworthy was the greater decline in rectal tem perature in lambs from high-Cu ewes (data pooled across chamber temperatures), in spite of their elevated BAT *UCP1* gene expression. Cold exposure drastically depleted BAT lipid regardless of prenatal Cu exposure, and the electron micrographs suggest that any remaining lipid in BAT after cold exposure resided primarily in membrane phospholipids. The data indicate that BAT lipid stores of all cold-exposed lambs had been depleted rapidly, leading to an inability to further generate heat via BAT nonshivering thermogenesis. Plasma NEFA concentration was lower in cold-exposed lambs, which is consistent with the depletion of BAT lipid stores seen by 48 h.

In conclusion, prenatal supplementation of Cu increased plasma T_3_ concentration and BAT *UCP1* expression, but this may have caused reduced tolerance to cold exposure. The results of this study suggest that the inability of lambs to maintain body temperatures during extended cold exposure is due to depletion of lipid stores in BAT. The data of the current study indicate that, if lambs are born at a time when ambient temperatures are near freezing, they have only limited ability to maintain body temperature via non-shivering, BAT thermogenesis. Conversely, lambs born in warm environments maintain their lipid stores, but rapidly lose capacity for BAT thermogenesis, as reflected by loss of *UCP1* expression by 48 h of age. Prenatal Cu supplementation delays the loss of *UCP1* expression, but that may exacerbate body heat loss during cold exposure. What could not be determined from the current study was the mechanism of action by which increasing maternal intake of Cu enhanced *UCP1* gene expression in BAT of the newborn lambs.

## Figures and Tables

**Table 1:** Ingredient and nutrient composition of experimental diets

Items	Low Cu	Medium Cu	High Cu
Ingredient (% as fed)			
Corn	38.99	38.98	38.98
Cottonseed hulls	34.68	34.68	34.68
Rice meal feed	18.97	18.97	18.97
Meat and bone meal	4.68	4.68	4.68
Cottonseed meal	1.50	1.50	1.50
Ca-SO_4_	0.53	0.53	0.53
Limestone	0.29	0.29	0.29
Salt	0.225	0.225	0.225
Vitamins A, D, E	0.124	0.124	0.124
Na-Mo	0.001	0.001	0.001
Cu-lysine	0	0.014	0.034
Nutrients (DM basis)			
DM (%)	89.0	89.0	89.0
CP (%)	12.3	12.3	12.3
ADF (%)	31.57	31.61	31.58
Ca (%)	0.96	0.96	0.96
P (%)	0.69	0.69	0.69
S (%)	0.28	0.28	0.28
Mo (ppm)	2.0	2.0	2.0
Cu (ppm)	3.25	10.43	20.73

**Table 2:** Growth and intake of ewes fed three levels of dietary copper

Items	Low Cu1)	Medium Cu1)	High Cu1)	SEM	p-values
Initial body weight (kg)	60.3	59.8	59.8	2.4	>0.25
Final body weight (kg)	78.1	75.7	76.3	2.7	>0.25
ADG (kg/d)	0.20	0.20	0.21	0.04	>0.25
DMI (kg/d)	1.78	1.69	1.67	0.04	0.19

SEM, standard error of the mean; ADG, average daily gain; DMI, dry matter intake (individual intake).

1) Low, medium, and high Cu: 3.2, 10, and 20 ppm Cu (DM basis), respectively.

**Table 3:** Plasma Cu, ceruloplasmin, and thyroid hormones in ewes and plasma Cu and ceruloplasmin in lambs following prenatal treatment with three levels of dietary copper

Items	Low Cu1)	Medium Cu1)	High Cu1)	SEM	p-values
Ewes					
Cu (μg/mL)	1.03	1.01	1.10	0.04	0.11
Ceruloplasmin (IU)	27.6	27.8	26.8	0.94	>0.25
Triiodothreonine (ng/dL)	196^b^	243^ab^	296^a^	19	<0.001
Thyroxine (μg/dL)	7.58^b^	10.58^ab^	12.23^a^	1.17	<0.01
Lamb (at birth)					
Cu (μg/mL)	0.41	0.35	0.39	0.04	>0.25
Ceruloplasmin (IU)	2.58	2.14	3.04	0.10	>0.25
Lamb (at 48 h)2)					
Cu (μg/mL)	0.38	0.32	0.24	0.04	>0.25
Ceruloplasmin (IU)3)	5.18^b^	7.59^ab^	9.88^a^	0.14	0.05

SEM, standard error of the mean; ADG, average daily gain; DMI, dry matter intake (individual intake).

1) Low, medium, and high Cu: 3.2, 10, and 20 ppm Cu (DM basis), respectively.

2) Chamber temperature had no effect on plasma Cu or ceruloplasmin activity (p>0.25), so data for these variables was pooled between warm and cold chamber temperatures.

3) Plasma ceruloplasmin activity increased with age for all prenatal Cu treatment groups (p<0.001).

a,b Values within a row with common superscripts do not differ (p>0.05).

**Table 4:** Lamb body weight, rectal temperature, and plasma hormone and metabolite concentrations following 48 h in warm or cold environmental chambers for lambs following prenatal exposure to three levels of dietary copper

Items	Dietary Cu concentration1)						SEM	p-values		

	Low Cu		Medium Cu		High Cu					
			
	Warm	Cold	Warm	Cold	Warm	Cold		Cu	Temp	Cu×Temp
Body weight (kg)	4.46	4.63	4.34	4.34	4.18	4.13	0.27	>0.25	>0.25	>0.25
Rectal temperature (°C)	39.0	37.8	39.0	36.9	38.0	35.9	0.3	0.03	<0.001	>0.25
Triiodothreonine (ng/dL)	145	251	111	208	103	187	13	0.08	<0.001	>0.25
Thyroxine (μg/dL)	6.18	8.77	5.99	8.58	5.77	9.56	0.38	>0.25	>0.25	>0.25
Cortisol (ng/dL)	2.58^c^	5.18^b^	2.14^c^	7.59^ab^	3.04^c^	9.88^a^	0.28	0.02	<0.001	0.05
Fatty acids (μEq/L)	457.7	244.6	403.5	267.1	504.1	263.7	22.3	>0.25	<0.001	>0.25
Glucose (mg/dL)	40.9	38.0	35.5	32.2	39.0	24.1	4.2	>0.25	>0.25	>0.25

SEM, standard error of the mean.

1) Low, medium, and high Cu: 3.2, 10, and 20 ppm Cu (DM basis), respectively; warm, 28°C, cold, 6°C temperature exposure.

a–c Values within a row with common superscripts do not differ (p>0.05).

**Table 5:** Brown adipose tissue mass, composition, norepinephrine and dopamine concentrations, and cytochrome c oxidase activity at 48 h of age in lambs following prenatal exposure to three levels of dietary copper

Brown adipose tissue item	Dietary Cu concentration1)						SEM	p-values		

	Low Cu		Medium Cu		High Cu					
			
	Warm	Cold	Warm	Cold	Warm	Cold		Cu	Temp	Cu×Temp
Mass (g)	19.4	11.6	20.2	10.9	17.7	9.5	0.96	0.22	<0.001	>0.25
Composition (mg/g BAT)										
Lipid	436.8	80.3	453.1	79.7	376.5	74.7	28.0	>0.25	<0.001	>0.25
Protein	93.6	134.9	97.5	132.7	96.7	128.8	6.1	>0.25	<0.01	>0.25
Moisture	454.8	751.6	427.7	744.0	465.2	748.2	27.7	>0.25	<0.01	>0.25
DNA	3.34^c^	4.14^b^	3.27^c^	4.41^b^	3.47^bc^	5.53^a^	0.20	<0.001	<0.001	<0.01
Mitochondrial protein (μg/g BAT)	23.58	38.53	25.34	33.63	23.76	37.26	2.70	>0.25	<0.01	>0.25
Cytochrome c oxidase activity (μmol/[min·g BAT])	49.2	160.9	60.4	157.0	53.5	145.5	12.5	>0.25	<0.01	>0.25
Norepinephrine (μg/g BAT)	1.45	0.97	1.19	0.74	0.95	0.82	0.13	0.07	<0.01	>0.25
Dopamine (μg/g BAT)	0.64^bc^	0.85^b^	0.67^bc^	0.83^b^	0.52^c^	1.12^a^	0.10	>0.25	<0.001	0.04

SEM, standard error of the mean; BAT, brown adipose tissue.

1) Low, medium, and high Cu: 3.2, 10, and 20 ppm Cu (DM basis), respectively; warm, 28°C, cold, 6°C temperature exposure.

a–c Values within a row with common superscripts do not differ (p>0.05).

## References

[1] Stott AW, Slee J (1985). The effect of environmental temperature during pregnancy on thermoregulation in the newborn lamb. Anim Prod.

[2] Cassard-Doulcier AM, Gelly C, Fox N (1993). Tissue-specific and beta-adrenergic regulation of the mitochondrial uncoupling protein gene: control by cis-acting elements in the 5′ flanking region. Mol Endocrinol.

[3] Leonard JL, Mellen SA, Larsch PR (1983). Thyroxine 5′-deiodinase activity in brown adipose tissue. Endocrinology.

[4] Masaki T, Yoshimatsu H, Kakuma T, Hidaka S, Kurokawa M, Sakata T (1997). Enhanced expression of uncoupling protein 2 gene in rat white adipose tissue and skeletal muscle following chronic treatment with thyroid hormone. FEBS Lett.

[5] Eales FA, Gilmour JS, Barlow RM, Small J (1982). Causes of hypothermia in 89 lambs. Vet Rec.

[6] Lukaski HC, Hall CB, Marchello MJ (1995). Body temperature and thyroid hormone metabolism of copper-deficient rat. J Nutr Biochem.

[7] National Research Council (1985). Nutrient requirements of sheep.

[8] Agricultural Research Council (1980). The nutrient requirement of ruminant livestock, Commonwealth Agricultural Bureaux.

[9] Martin GS, Carstens GE, King MD, Eli AG, Mersmann HJ, Smith SB (1999). Metabolism and morphology of brown adipose tissue from Brahman and Angus newborn calves. J Anim Sci.

[10] Chen C, Carstens GE, Gilbert CD (2007). Dietary supplementation of high levels of saturated and monounsaturated fatty acids to ewes during late gestation reduces thermogenesis in newborn lambs by depressing fatty acid oxidation in perirenal brown adipose tissue. J Nutr.

[11] Cannon B, Lindberg O (1979). Mitochondria from brown adipose tissue: isolation and properties. Methods Enzymol.

[12] Billington CT, Bartness TJ, Briggs J, Levine AS, Morley JE (1987). Glucagon stimulation of brown adipose tissue growth and thermogenesis. Am J Physiol.

[13] Burton KA (1956). A study of the conditions and mechanism of the diphenylamine reaction for the colorimetric estimation of deoxyribonucleic acid. Biochem J.

[14] Markwell MN (1978). A modification of the Lowry procedure to simplify protein determination in membrane and lipoprotein samples. Anal Biochem.

[15] Houchin OB (1958). A rapid colorimetric method for the quantitative determination of copper oxidase activity (ceruloplasmin). Clin Chem.

[16] Clarke L, Bird JA, Lomax M, Symonds ME (1996). Effect of β_3_-adrenergic agonist (Zeneca D7114) on thermoregulation in near-term lambs delivered by cesarean section. Pediatr Res.

[17] SAS Institute Inc (1985). SAS user’s guide: basics.

[18] Kincaid RL, White CL (1988). The effects of ammonium tetrathiomolybdate intake of tissue copper and molybdenum in pregnant ewes and lambs. J Anim Sci.

[19] Alexander G (1978). Quantitative development of adipose tissue in foetal sheep. Aust J Biol Sci.

[20] Landis MD, Carstens GE, McPhail EG (2002). Ontogenic development of brown adipose tissue in Angus and Brahman fetal calves. J Anim Sci.

[21] Pope M, Budge H, Symonds ME (2014). The developmental transition of ovine adipose tissue through early life. Acta Physiol.

[22] Giralt M, Casteilla L, Viñas O (1989). Iodothyronine 5′-deiodinase activity as an early event of prenatal brown-fat differentiation in bovine development. Biochem J.

[23] de Jesus LA, Carvalho SD, Ribeiro MO (2001). The type 2 iodothyronine deiodinase is essential for adaptive thermogenesis in brown adipose tissue. J Clin Invest.

[24] Petrovic N, Cvijic G, Davidovic V (2003). Thyroxine and tri-iodothyronine differently affect uncoupling protein-1 content and antioxidant enzyme activities in rat interscapular brown adipose tissue. J Endocrinol.

[25] Henry BA, Loughnan R, Hickford J, Young IR, St John JC, Clarke I (2015). Differences in mitochondrial DNA inheritance and function align with body conformation in genetically lean and fat sheep. J Anim Sci.

[26] Bianco AC, Carvalho SD, Carvalho CR, Rabelo R, Moriscot AS (1998). Thyroxine 5′-deiodination mediates norepinephrine-induced lipogenesis in dispersed brown adipocytes. Endocrinology.

[27] Wu SY, Merryfield ML, Polk DH, Fisher DA (1990). Two pathways for thyroxine 5′-monodeiodination in brown adipose tissue in fetal sheep: ontogenesis and divergent responses to hypothyroidism and 3,5,3′-triiodothyronine replacement. Endocrinology.

[28] Geloen A, Collet AJ, Guay G, Bukowiecki LJ (1988). Beta-adrenergic stimulation of brown adipocyte proliferation. Am J Physiol.

[29] Smith SB, Carstens GE, Burrin D, Mersmann HJ (2005). Ontogeny and metabolism of brown adipose tissue in livestock species. Biology of metabolism in growing animals.

[30] Trayhurn PM, Thomas MEA, Keith JS (1993). Postnatal development of uncoupling protein, uncoupling protein mRNA, and GLUT4 in adipose tissues of goats. Am J Physiol.

